# A Technique for Serial Collection of Cerebrospinal Fluid from the Cisterna Magna in Mouse 

**DOI:** 10.3791/960

**Published:** 2008-11-10

**Authors:** Li Liu, Karen Duff

**Affiliations:** Department of Pathology, Columbia University

## Abstract

Alzheimer's disease (AD) is a progressive neurodegenerative disease that is pathologically characterized by extracellular deposition of β-amyloid peptide (Aβ) and intraneuronal accumulation of hyperphosphorylated tau protein. Because cerebrospinal fluid (CSF) is in direct contact with the extracellular space of the brain, it provides a reflection of the biochemical changes in the brain in response to pathological processes. CSF from AD patients shows a decrease in the 42 amino-acid form of Aβ (Aβ42), and increases in total tau and hyperphosphorylated tau, though the mechanisms responsible for these changes are still not fully understood.  Transgenic (Tg) mouse models of AD provide an excellent opportunity to investigate how and why Aβ or tau levels in CSF change as the disease progresses. Here, we demonstrate a refined cisterna magna puncture technique for CSF sampling from the mouse. This extremely gentle sampling technique allows serial CSF samples to be obtained from the same mouse at 2-3 month intervals which greatly minimizes the confounding effect of between-mouse variability in Aβ or tau levels, making it possible to detect subtle alterations over time. In combination with Aβ and tau ELISA, this technique will be useful for studies designed to investigate the relationship between the levels of CSF Aβ42 and tau, and their metabolism in the brain in AD mouse models. Studies in Tg mice could provide important validation as to the potential of CSF Aβ or tau levels to be used as biological markers for monitoring disease progression, and to monitor the effect of therapeutic interventions. As the mice can be sacrificed and the brains can be examined for biochemical or histological changes, the mechanisms underlying the CSF changes can be better assessed. These data are likely to be informative for interpretation of human AD CSF changes.

**Figure:**
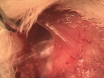


## Protocol

**Pulling the glass capillary tube**

1. The glass capillary tube is purchased from The Sutter Instrument Inc (Borosilicate glass, B100-75-10).
2. Pull the capillary tubes on a Sutter P-87 Flaming micropipette puller, with the heat index set at 300 and the pressure index set at 330.
3. Trim the tip of the glass capillary tube with scissors, so that the tapered tip has an inner diameter of about 0.5 mm.

**Cisterna magna puncture technique for CSF**

CSF samples are taken from the cisterna magna (Figure 1) using a method that was published previously ^1^.
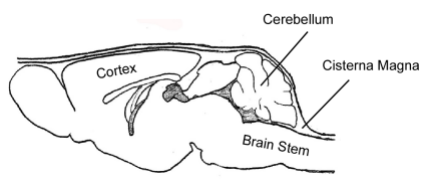
**Figure 1**

1. The mice are anesthesized by Ketamine (100mg/kg) and xylazine (10mg/kg), administered intraperitoneally. During the time of anesthesia induction, the mice are kept in a 37^o^C incubator.

2. The skin of the neck is shaved, and the mouse is then placed prone on the stereotaxic instrument with direct contact of a heating pad. A rectal temperature probe is inserted into the rectum so that the heat produced by the heating pad is adjusted in response to the changes of body temperature. The head is secured with the head adaptors. The surgical site is swabbed with 10% povidone *iodine, followed by 7*0% ethanol (repeat 3 times), and a sagittal incision of the skin is made inferior to the occiput.

3. Under the dissection microscope, the subcutaneous tissue and muscles (*m. biventer cervicis* and *m. rectus capitis dorsalis major*) are separated by blunt dissection with forceps. A pair of microretractors is used to hold the muscles apart.

4. The mouse is laid down so that the head forms a nearly 135^o^ angle with the body.

5. Under the dissection microscope, the dura mater of the cisterna magna appears as a glistening and clear reverse triangle through which the medulla oblongata and a major blood vessel (arteria dorsalis spinalis), and the CSF space are visible (Figure 2).
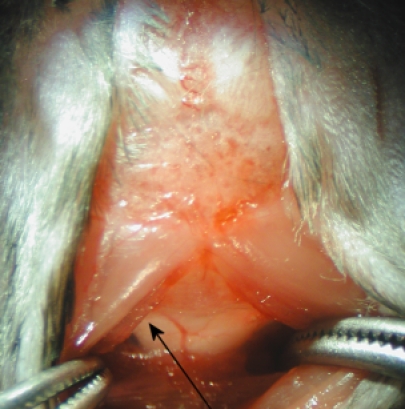
**Figure 2**

6. The dura mater is blotted dry with sterile cotton swab. Penetrate the capillary tube into the citerna magna through the dura mater, lateral to the arteria dorsalis spinalis (Figure 2). Following a noticeable change in resistance to the capillary tube insertion, the CSF flows into the capillary tube.

7. Carefully remove the capillary tube, and connect it to a 3 ml syringe through a polyethylene tubing that has a 1 mm internal diameter. Inject the CSF into a pre-marked 0.5 ml eppendorf tube, and freeze the tube immediately on dry ice and then transfer it into a -80°C freezer.

*8. *After CSF sampling, the muscles are re-aligned, and skin is sutured (4-0, Ethicon, Johnson & Johnson). About 1 ml of 0.9% NaCl is injected subcutaneously to prevent de-hydration. The mouse is kept at the incubator to maintain body temperature until it recovers; the weight of the mouse is monitored 1 day, and 1 week after the surgery.

**Results and Discussion**

We described a highly reliable protocol for serial sampling of CSF from mice without detectable plasma contamination.

1. The whole procedure usually takes 10 min per mouse (including anesthesia). The volume of CSF obtained is dependent on the mouse strains. In the PS/APP double Tg mice we used ^2^, the average volume is about 5 μl (3 to 7 μl), while the P301L (JNPL3) mice ^3^ give a yield of about 10 μl- 15 μl. For serial sampling, a maximum of 7-8 μl can be safely taken each time at an interval of 2-3 months.

2. During the surgery, it's important to position the head and the body of the mouse properly on the stereotaxic frame so that the dura mater of cisterna magna can be exposed sufficiently. Carefully avoid the blood vessels when penetrating the dura mater with the capillary tube to prevent contamination from plasma proteins, which can be monitored in the CSF sample by immunoblotting for ApoB ^4^.

3. Minimizing the tissue damage during each surgery is important, as tissue conglutination can increase the difficulty by predisposing bleeding for the following sampling.

4. The body temperature needs to be well maintained during the surgery because hypothermia caused by anesthesia can significantly affect the phosphorylated tau levels in the brain very rapidly ^5^.

5. The Ab ELISA protocol and antibodies that we used were described in detail in Refolo et al. ^6^. Two μl of CSF from the PS/APP mice will give satisfactory results when diluted 1:50-1:60. For the tau ELISA, 4-5 μl CSF from P301L (JNPL3) mice will be sufficient for the detection of total tau, or *tauphosphor*ylated at threonine*231* (Kits are purchased from Bio-Source).

6. This technique can be applied to other mouse models of neurological diseases, for which a small volume of CSF is satisfactory for desired assays.

## Discussion

We described a highly reliable protocol for serial sampling of CSF from mice without detectable plasma contamination.

1. The whole procedure usually takes 10 min per mouse (including anesthesia).  The volume of CSF obtained is dependent on the mouse strains. In the PS/APP double Tg mice we used2, the average volume is about 5 μl (3 to 7 μl), while the P301L (JNPL3) mice 3 give a yield of about 10 μl- 15 μl.  For serial sampling, a maximum of 7-8 μl can be safely taken each time at an interval of 2-3 months.

2. During the surgery, it is important to position the head and the body of the mouse properly on the stereotaxic frame so that the dura mater of cisterna magna can be exposed sufficiently. Carefully avoid the blood vessels when penetrating the dura mater with the capillary tube to prevent contamination from plasma proteins, which can be monitored in the CSF sample by immunoblotting for ApoB ^4^.

3. Minimizing the tissue damage during each surgery is important, as tissue conglutination can increase the difficulty by predisposing bleeding for the following sampling.

4. The body temperature needs to be well maintained during the surgery because hypothermia caused by anesthesia can significantly affect the phosphorylated tau levels in the brain very rapidly ^5^.

5. The Ab ELISA protocol and antibodies that we used were described in detail in Refolo et al. ^6^. Two μl of CSF from the PS/APP mice will give satisfactory results when diluted 1:50-1:60. For the tau ELISA, 4-5 μl CSF from P301L (JNPL3) mice will be sufficient for the detection of total tau, or tau phosphorylated at threonine 231 (Kits are purchased from Bio-Source).

6. This technique can be applied to other mouse models of neurological diseases, for which a small volume of CSF is satisfactory for desired assays.
